# A sound coding strategy based on a temporal masking model for cochlear implants

**DOI:** 10.1371/journal.pone.0244433

**Published:** 2021-01-08

**Authors:** Eugen Kludt, Waldo Nogueira, Thomas Lenarz, Andreas Buechner

**Affiliations:** 1 Department of Otolaryngology, Medical University of Hannover, Hanover, Germany; 2 Hearing4all, Oldenburg, Germany; Universidad de Salamanca, SPAIN

## Abstract

Auditory masking occurs when one sound is perceptually altered by the presence of another sound. Auditory masking in the frequency domain is known as simultaneous masking and in the time domain is known as temporal masking or non-simultaneous masking. This works presents a sound coding strategy that incorporates a temporal masking model to select the most relevant channels for stimulation in a cochlear implant (CI). A previous version of the strategy, termed psychoacoustic advanced combination encoder (PACE), only used a simultaneous masking model for the same purpose, for this reason the new strategy has been termed temporal-PACE (TPACE). We hypothesized that a sound coding strategy that focuses on stimulating the auditory nerve with pulses that are as masked as possible can improve speech intelligibility for CI users. The temporal masking model used within TPACE attenuates the simultaneous masking thresholds estimated by PACE over time. The attenuation is designed to fall exponentially with a strength determined by a single parameter, the temporal masking half-life *T*_*½*_. This parameter gives the time interval at which the simultaneous masking threshold is halved. The study group consisted of 24 postlingually deaf subjects with a minimum of six months experience after CI activation. A crossover design was used to compare four variants of the new temporal masking strategy TPACE (*T*_*½*_ ranging between 0.4 and 1.1 ms) with respect to the clinical MP3000 strategy, a commercial implementation of the PACE strategy, in two prospective, within-subject, repeated-measure experiments. The outcome measure was speech intelligibility in noise at 15 to 5 dB SNR. In two consecutive experiments, the TPACE with *T*_*½*_ of 0.5 ms obtained a speech performance increase of 11% and 10% with respect to the MP3000 (*T*_*½*_ = 0 ms), respectively. The improved speech test scores correlated with the clinical performance of the subjects: CI users with above-average outcome in their routine speech tests showed higher benefit with TPACE. It seems that the consideration of short-acting temporal masking can improve speech intelligibility in CI users. The half-live with the highest average speech perception benefit (0.5 ms) corresponds to time scales that are typical for neuronal refractory behavior.

## Introduction

Cochlear implants restore functional hearing in subjects with severe to profound hearing loss, but despite the enormous success and recent advancements, users of these devices still have extensive problems to understand speech in challenging environments such as background noise or reverberation [1, 2]. Generally, CIs consist of an externally worn sound processor and an implant connected to an electrode array carrying up to 22 electrode contacts. The electrode array is positioned inside the scala tympani for the purpose of electrically stimulating neural elements in the cochlear modiolus. The sound processor is responsible for decomposing the audio signal into different frequency bands or channels and determining the signal amplitudes in these channels. Each of these channels corresponds to one intra-cochlear electrode contact to which appropriate electrical currents proportional to the band-pass amplitudes are applied. A detailed and comprehensive description of this process and the different sound coding strategies can be found in [3, 4].

One factor that restricts speech intelligibility is the relatively crude interface between the CI electrodes and the auditory nerve (e. g. [5, 6]). Electrical stimulation inside the fluid-filled cochlea leads to significant current spread reducing the number of independent channels in the cochlea. As reviewed in Richter et al. [7], only 4 to 7 intracochlear electrode contacts of today’s implants can be considered independent, while normal hearing subjects can process 30–50 independent channels. As a result, the amount of independent information, which can be conveyed towards higher processing stages in the auditory system is significantly reduced.

One possible approach to improve the speech intelligibility performance of the patients is to identify perceptually relevant signal components and enhance their presentation [8]. The rationale here is to reduce the number of stimuli being sent through the electrode-nerve-interface, implicitly also reducing channel interaction due to the sparser coding of the acoustic signal. Forerunner strategies of this principle have been the so called n-of-m strategies. The n-of-m strategy estimates the envelope amplitude for each of the m bandpass filters in the system (one for each channel), and selects the n channels (defined as number of maxima in the fitting parameters) with the largest amplitude for stimulation. Therefore, in any period only n channels execute stimulation pulses to their respective electrode contact. Especially in the earlier days of cochlea implantation, this simple maxima selection was particularly used to increase the temporal resolution of selected channels, as the implant hardware typically was too slow to stimulate all intra cochlear electrodes for each processing frame [9].

A more recent approach for identification of perceptually relevant signal components is the use of auditory masking models. Auditory masking is a phenomenon under which one sound becomes less audible under the presence of another sound close in frequency (simultaneous masking) or in time (temporal masking) with high enough level. Auditory masking levels have been determined empirically through psychoacoustic hearing experiments [10–12] and can be used for extraction of the most meaningful components in an audio signal. The Psychoacoustic Advanced Combination Encoder (PACE) strategy uses such a psychoacoustic model (see section below for more details) of simultaneous masking to improve perceptual relevance of channel selection [8, 13].

The results with PACE, which was clinically released under the name of MP3000, were encouraging [14]. It was possible to reduce the number of stimulated electrodes from 6–14 to 4–6 channels with MP3000 and preserve the same level of speech intelligibility as with the clinical default strategy Advanced Combination Encoder (ACE) on the Cochlear Nucleus 24 device. MP3000 successfully reduced the energy consumption and provided 24% longer battery time due to the reduced number of stimuli, the performance of the patients however was not further improved over that with the ACE strategy [14]. We have designed a novel CI coding strategy that adds a model of temporal masking to the PACE. The new strategy, termed the Temporal Psychoacoustic Advanced Combination Encoder (TPACE) follows the principle of removing unnecessary information to further take load off the limited electrode-nerve interface. The algorithm for channel selection was extended by incorporating a temporal masking model additionally to the simultaneous masking concept (see section below for more details).

Psychoacoustic experiments have shown that the amount of temporal masking increases with a shorter masker-signal interval and decreasing frequency separation [15]. Although simultaneous masking is predominantly used in the field of perceptual audio coding, temporal masking–particularly post-masking or forward masking—does also play a significant role and is implemented in many data compression algorithms for audio signals [16]. For practical reasons, we only considered forward masking in our implementation, as the use of backward masking is hardly possible in real-time audio processing. We focused on the peripheral effect of the absolute and relative refractory recovery of spiral ganglion neurons with half-lives equal or below 1.1 ms, which covered the range of previously reported recovery function time constants [17, 18]. A motivation to incorporate temporal masking is that stimulation before the completion of the auditory nerve recovery may be of little use and could even lead to a loss of information transmitted towards the auditory nerve.

Our goal was to compare the speech intelligibility outcomes of the new TPACE with respect to the existing MP3000 strategy in CI subjects. Furthermore, we evaluated whether there is a relationship between the refractoriness of the auditory nerve fibers (ANFs) measured by Neural Response Telemetry (NRT) and different slopes of masking decay used in the TPACE implementation. It is known that there is individual variation in refractory period across CI subjects and a relationship between the masking half-lives and the neural refractory period might potentially allow for a patient-specific individualization of the temporal masking behavior in order to maximize the benefit with the new TPACE strategy.

Other approaches also use auditory models in order to improve the speech recognition and the perceived quality by simulation of the hearing system properties. The Stimulation based on Auditory Modeling (SAM) coding strategy consists of an auditory model and a coder. The auditory model simulates the function of the peripheral ear, nonlinear mechanical filtering and mechano-electrical transduction [19, 20]. It accounts for temporal masking effects by a phasic response that enhances stimulus onsets. The auditory model of the SAM strategy mimics also other properties of the auditory system up to the level of the ribbon synapses: realistic cochlear delays, compression and phase-locking. Subsequent processing steps in the SAM strategy are performed by the coder part. The SAM coder maps the output of the SAM auditory model to electrode stimulations. The bio-inspired coding (BIC) strategy accounts for phenomena that are introduced to the auditory system by electrical stimulation of the inner ear (e.g. spread of excitation) [21]. The electric stimulation of ANFs differs fundamentally from synaptic transmission in the organ of Corti. The responses of ANFs on electrical stimulation show the effects of absolute refractoriness (time interval directly after an action potential with zero probability for generation of the next action potential), relative refractoriness (time interval after absolute refractoriness with rising probability to generate the next action potential), facilitation (increase of neuron excitability for short interpulse intervals) and adaptation (decrease of neuron excitability upon repeated action potential generation) [22]. The BIC strategy explicitly models all these effects and also includes a simultaneous masking model in order to reduce channel interaction [21], as described in the motivation for the development of the PACE strategy above.

Both SAM and BIC strategies break up the boundaries of conventional framewise stimulation paradigms and introduce one by one stimulation, in which the stimulation of each pulse is calculated individually, taking the effects of preceding stimulations into account and allowing for individual interpulse intervals. Both the SAM and BIC strategies mimic several properties of the auditory system causing substantial changes in the stimulation patterns. Our approach with TPACE, in contrast, was to mainly focus on the temporal masking effects occurring in the auditory system. The temporal integrator processing strategy (TIPS) also modeled temporal masking in CI explicitly by introducing a sliding temporal integrator into a conventional sound processing chain [23]. The temporal integrator was applied on individual channels and was used to remove masked pulses without interaction to the neighboring channels, following a similar idea as with the TPACE sound coding strategy. The TIPS was compared to a custom made sound coding strategy with a reduced number of active electrodes that did not perform channel selection contrarily to the clinical strategy of the study participants. In contrast, the current study investigates the effect of adding temporal masking for channel selection on speech understanding performance using a clinically available n-of-m-type sound coding strategy as a reference.

## Materials and methods

### Study participants

The study groups for two consecutive experiments consisted of 12 postlingually deaf subjects each. All subjects had a minimum of six-month experience with the Nucleus CI system (Table 1) and used either the ACE or MP3000 strategy in daily life and were at least able to understand 50% speech in quiet.

**Table 1:** Subject demographics.

Subject	Age [years]	Duration of deafness at time of study [years]	Reason for deafness	Implant Type	CI usage [years]		Clinical Outcome (HSM) [%]		clinical map				
					ipsi	contra	quiet	10 dB	strategy	stim. rate [pps]	# max	# el.	Tested SNR [dB]
1.1	57	4	Sudden hearing loss	RE-24 CA	4	-	100	50	ACE	900	8	22	10
1.2	72	12	Unknown	RE-24 CA	4	10	99	31	MP3000	900	5	22	5
1.3	75	3	Morbus Meniere	RE-24 CA	3	-	100	45	MP3000	900	5	22	5
1.4	71	2	Explosion trauma	CI512	1	-	97	24	ACE	900	8	22	10
1.5	63	25	Unknown	RE-24 CA	3	20	58	NM	MP3000	900	5	21	15
1.6	32	10	Sudden hearing loss	RE-24 CA	4	10	100	58	ACE	1200	8	22	5
1.7	62	0	Sudden hearing loss	RE-24 CA	3	0,4	100	61	MP3000	900	5	22	10
1.8	70	2	Unknown	CI512	2	1	92	11	ACE	900	8	22	10
1.9	53	10	Unknown	CI512	2	1	93	0	ACE	900	8	22	10
1.10	28	1	Unknown	CI512	1	-	98	8	ACE	900	8	22	10
1.11	33	13	Unknown	RE-24 CA	4	7	100	52	ACE	900	8	22	5
1.12	64	12	Sudden hearing loss	RE-24 CA	7	4	80	20	ACE	500	10	20	15
2.1	19	8	Unknown	RE-24 CA	8	-	100	77	MP3000	1200	5	22	5
2.2	72	10	Unknown	CI512	4	5	91	15	ACE	900	8	22	10
2.3	77	12	Sudden hearing loss	CI512	4	12	85	0	ACE	900	8	22	15
2.4	53	6	Unknown	RE-24 CA	6	-	99	34	ACE	900	8	22	10
2.5	49	18	Meningitis	CI512	4	4	90	16	ACE	900	8	22	15
2.6	66	8	Sudden hearing loss	RE-24 CA	8	7	100	83	ACE	900	8	22	5
2.7	63	6	Unknown	RE-24 CA	5	4	86	8	ACE	900	8	22	15
2.8	78	1	Unknown	RE-24 CA	1	0,5	76	58	MP3000	900	5	22	10
2.9	72	18	Otitis Media	RE-24 CA	5	10	91	50	ACE	900	8	22	10
2.10	26	7	Pendred syndrome	RE-24 CA	7	-	97	8	MP3000	900	8	22	10
2.11	69	9	Sudden hearing loss	RE-24 CA	6	12	99	75	MP3000	900	5	22	5
2.12	65	11	Mumps	RE-24 CA	10	-	100	72	MP3000	900	6	22	5

stim. rate, stimulation rate; # max, maximal number of stimulated channels; # el., number of active electrodes; pps, pulses per second.

### Review of the electrode selection in the MP3000 strategy

**Fig 1:**
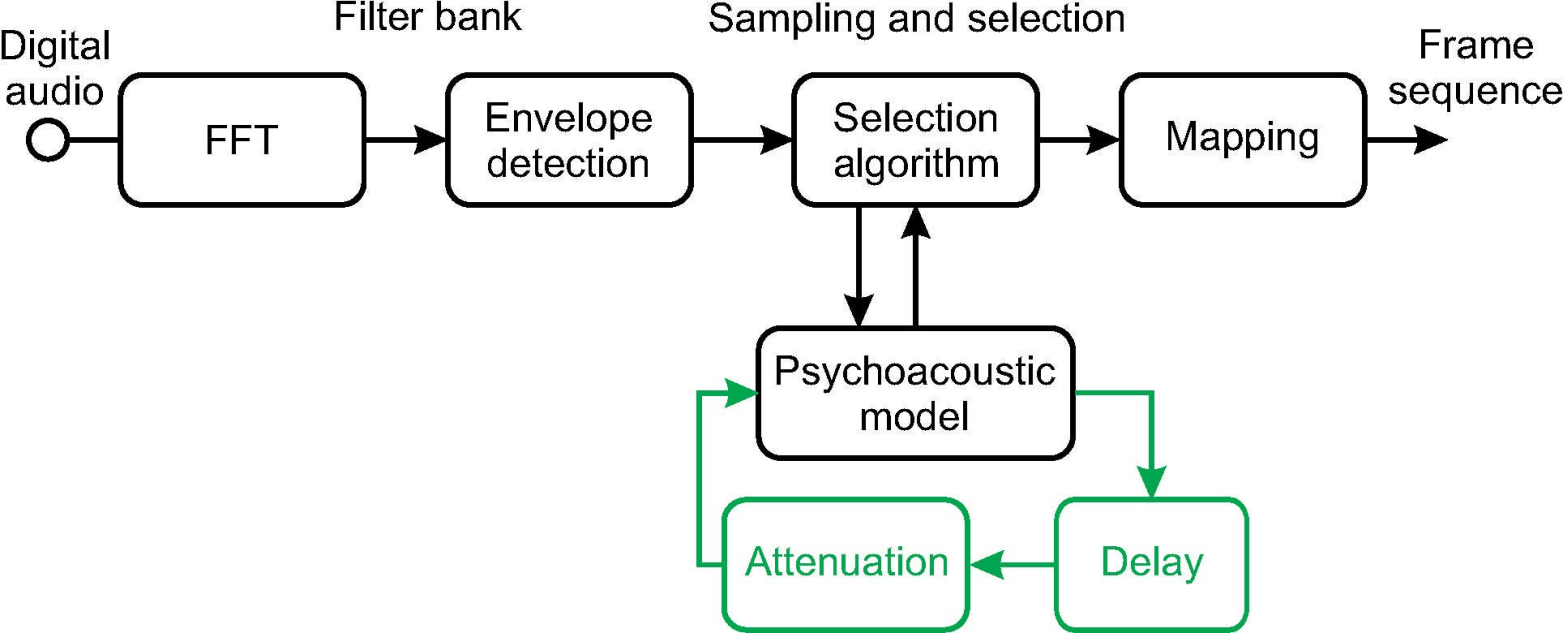
Block diagram illustrating the PACE (black) and TPACE (green) strategy.

The channel selection algorithm of the MP3000 strategy incorporates a psychoacoustic model that considers simultaneous masking to select channels for stimulation. Details can be found in [13], but a brief description of the basic principle of the PACE/MP3000 simultaneous masking model shall be given here. Implementation of the strategy has been done using the NIC (Nucleus Implant Communicator) development environment Version 2. A block diagram illustrating the MP3000 coding strategy is presented in Fig 1(in black). The digital audio signal, sampled at Fs = 16 kHz, is analyzed in frames of 128 samples and converted into the frequency domain using a fast fourier transform (FFT). Next, the amplitude envelope in each of the 22 frequency bands (z = 1,…,M) is computed combining the energy of the according FFT bins. The channel selection algorithm is then applied iteratively until the desired number of channels is reached, as follows: For the first channel selection, simply the channel with the highest amplitude with respect to the threshold of audibility is picked for stimulation, as there is obviously no simultaneous masking across channels beforehand. For the selection of the remaining channels within the same processing frame, the simultaneous masking model is being used, picking the channel affected by the least amount of masking. After each channel selection, the masking profile, i.e. the threshold of perception across all channels, is updated accordingly, obviously based on the channels which have already been selected. When all channels of one processing frame have been selected, the masking profile is set to zero and the process of channel selection starts from scratch.

### Implementation of TPACE strategy

TPACE introduces forward masking by carrying over the final masking profile from the preceding processing frame, instead of starting from scratch, i.e. the threshold of perception from frame n does not start from zero, but inherits the attenuated simultaneous masking thresholds from frame *f*-1 for all 22 channels (Fig 1, green). Subsequently, for the selection of the first channel in frame *f*, the channel with the strongest signal above the inherited masking threshold is selected. After that, similar to the MP3000 strategy, the masking profile is updated, considering the already selected channels in frame *f* and the attenuated masking thresholds of frame *f*-1. The process of channel selection is then repeated until the desired number of channels has been reached.

**Fig 2:**
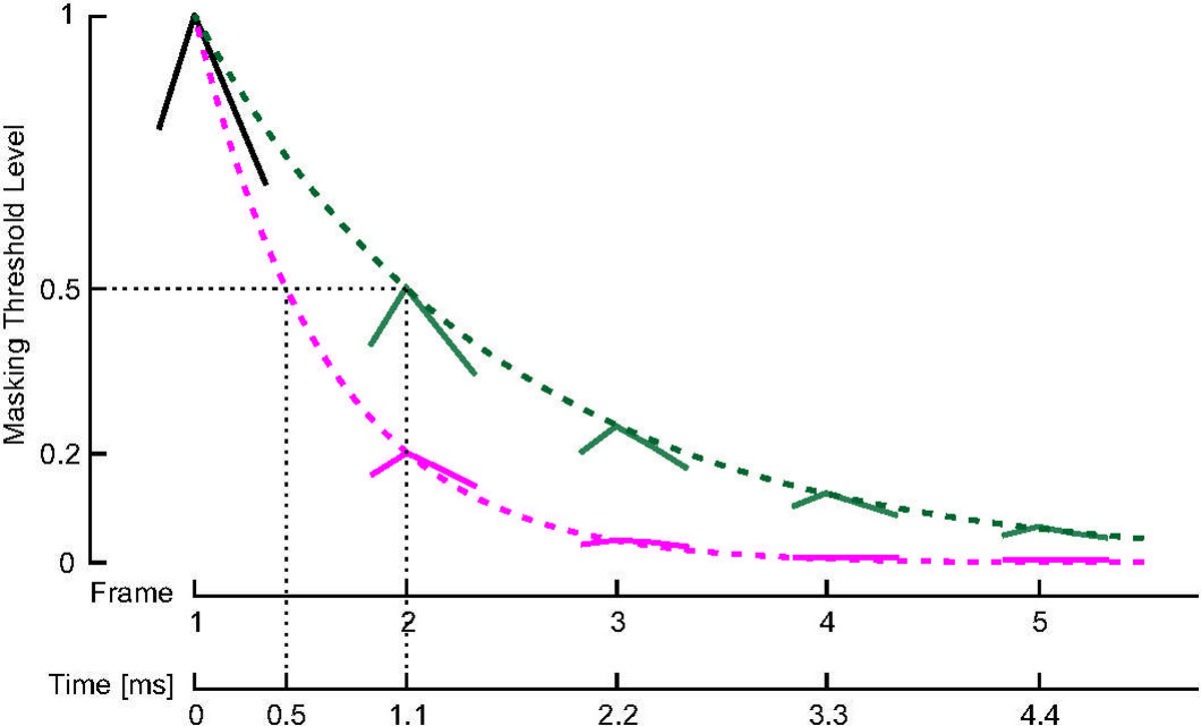
Model example of exponential decay of temporal masking thresholds (normalized to amplitude of 1) at two different temporal masking half-lifes *T*_*½*_ (0.5 ms in magenta and 1.1 ms in green). The stimulation rate in this model example is 900 pps. At this stimulation rate, *T*_*½*_ of 0.5 ms or 1.1 ms leads to a decrease of the temporal masking threshold by 50% or 20% in the following frame, respectively.

The strength of temporal masking in TPACE is determined by the temporal masking half-life *T*_*½*_ (Fig 2). This time constant indicates after which time the strength of the temporal masking has decreased to half of the amount of the initial masking at the time *T*_*0*_. As in TPACE the final masking profile of the preceding frame is being carried over (see above), the masking profile at the time *T*_*0*_ would exactly resemble the masking profile of the preceding frame (after selection of the last maximum). The goal is to make the slopes shallower across time such that the effect of the masking on selected channels gets reduced. This is implemented applying a factor F in the dB domain. According Nogueira et al. [13] Eq 1 the masking level in each channel z and frame *f* denoted by L_Tf_(z) in dB:

$$L_{T_{f}}(z)=F\cdot L_{T_{f-1}}(z).$$ (1)

To initialize the thresholds for a given frame, the attenuation factor *F*, which is applied to the masking threshold of the preceding frame, can be calculated from the stimulation rate (1/*t*) where *t* denotes the time variable in seconds and temporal masking half-life *T*_*½*_ using the following equation:

$$F=2^{-\frac{t}{T_{½}}}.$$ (2)

**Fig 3:**
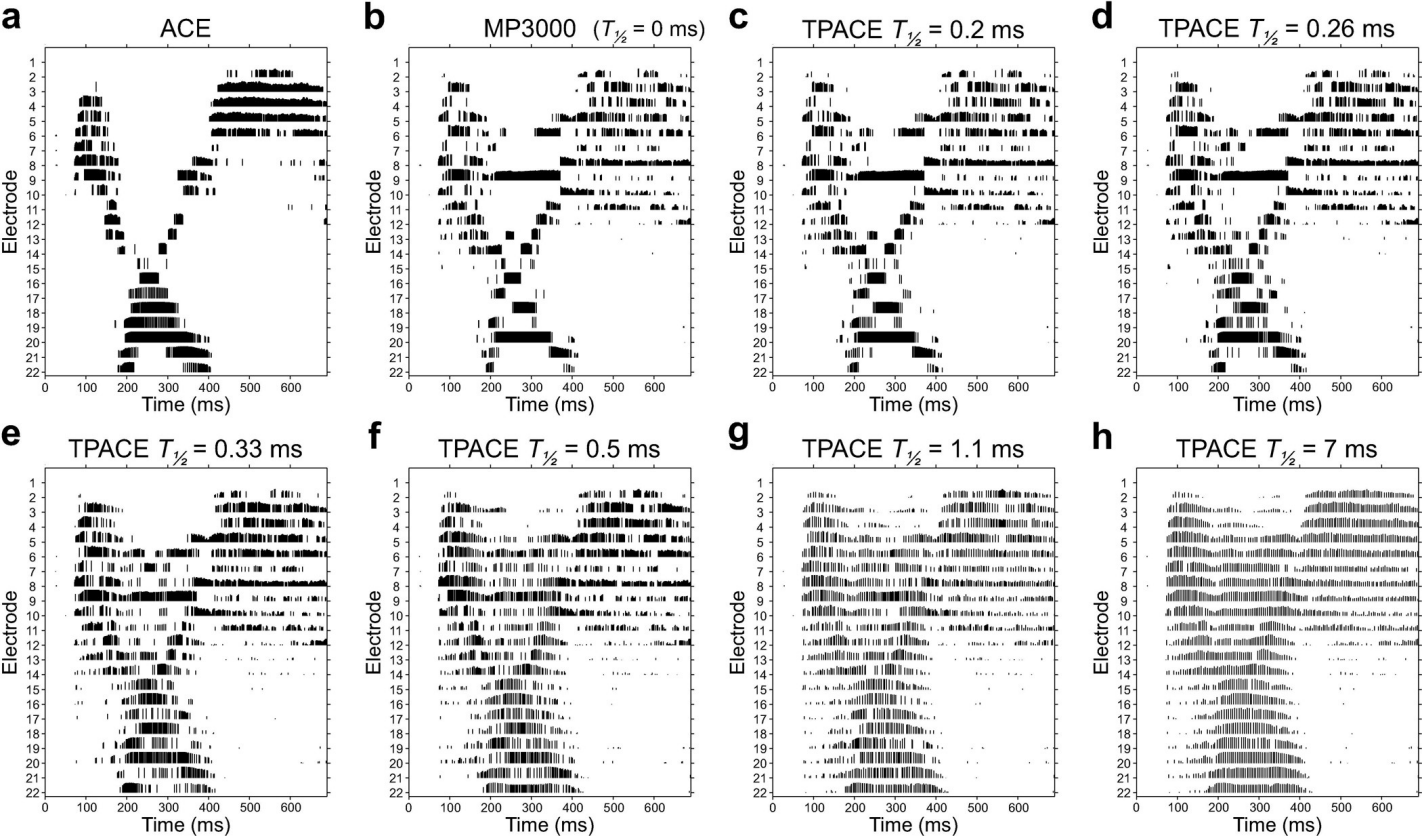
Stimulation patterns (electrodograms) for ACE (a), MP3000 (b) and TPACE with different values of the half-life value *T*_*½*_ (c-h). Current across time and electrode. Each vertical line represents the amplitude of a biphasic pulse in clinical units. Number of maxima was set to 5 in all stimulations, leading to maximum of five pulses in the same time frame. The acoustic input is an audio sample containing the word “choice”.

The effect of *T*_*½*_ on the channel selection is presented in the electrodograms of Fig 3. Short *T*_*½*_ cause a fast decay of the temporal masking. For values of 0.2 ms (Fig 3C) and below, the channel selection within the psychoacoustic model is hardly influenced by temporal masking, leading to an electrodogram that resembles the one of the MP3000 sound coding strategy (Fig 3B). With longer *T*_*½*_ values, temporal masking has more and more effect on the channel selection and the differences to the MP3000 algorithm become clearly visible at *T*_*½*_ = 0.5 ms (Fig 3F). Values of *T*_*½*_ above 1.1 ms further increase the impact of temporal masking, preventing the selection of the same electrode for stimulation in the directly following frame and thereby leading to strong redistribution of stimulation to regions with very low signal amplitudes (Fig 3H).

Therefore, *T*_*½*_ values of 0.5 and 1.1 ms were selected for the first evaluation of speech intelligibility in CI users. Furthermore, two additional *T*_*½*_ values of 0.4 and 0.8 ms were evaluated in a follow-up experiment.

### Evaluation of TPACE in acute streaming experiments

The TPACE algorithm was implemented using the Nucleus MATLAB Toolbox (Cochlear Ltd., Sydney, Australia) and the NIC research interface (Cochlear Ltd., Sydney, Australia) was used for two acute streaming experiments using the L34 research speech processor (Cochlear Ltd., Sydney, Australia). The signals were streamed from a PC directly to the implant.

The speech intelligibility of the subjects was measured using Hochmair-Schulz-Moser (HSM) sentence test [24]. A list of the HSM sentence test contains 106 words in 20 everyday sentences and were mixed at a constant signal to noise ratio (SNR) of 5, 10 and 15 dB with speech shaped stationary noise (“Comité Consultatif International Télégraphique et Téléphonique”, CCITT, according to ITU-T Rec. G.227 [11/88] conventional telephone signal). The same test in quiet and at 10 dB SNR was also used during clinical routine follow-up visits of these subjects.

In the first experiment, twelve subjects (P1.1 to P1.12, Table 1) were tested within one single session in three conditions: TPACE using *T*_*½*_ of 0.5, 1.1 ms and the original MP3000 algorithm. In a second experiment, twelve other subjects (P2.1 to P2.12, Table 1) were tested in a single session with HSM sentences in noise in five conditions: TPACE using *T*_*½*_ of 0.4, 0.5, 0.8, 1.1 ms and the original MP3000 algorithm.

Subjects who used the ACE strategy in their daily life were fitted with MP3000. This was done by globally increasing the comfort levels of electric stimulation (C-level) across all electrodes while life speech was presented to the subject until a comfortable loudness perception was reached. All streaming experiments were performed with 900 pps and 5 maxima. In order to avoid ceiling effects, a pilot testing with one list of the HSM sentence test at 10 dB SNR was performed. Subjects that scored more than 80% or less than 20% were tested at 5 dB SNR or 15 dB SNR respectively (Table 1, tested SNR). A single-subject repeated-measure design with two lists in an ABCDE-EDCBA testing sequence was used. Each subject served as her or his own control. Statistical analysis of the speech performance was performed with the non-parametric Quade Test [25]. Regression of the speech performance difference between TPACE and the MP3000 with demographic parameters (i.e. age, duration of deafness, CI usage duration, clinical performance) was performed using a least squares linear model.

### Neural response telemetry

**Fig 4:**
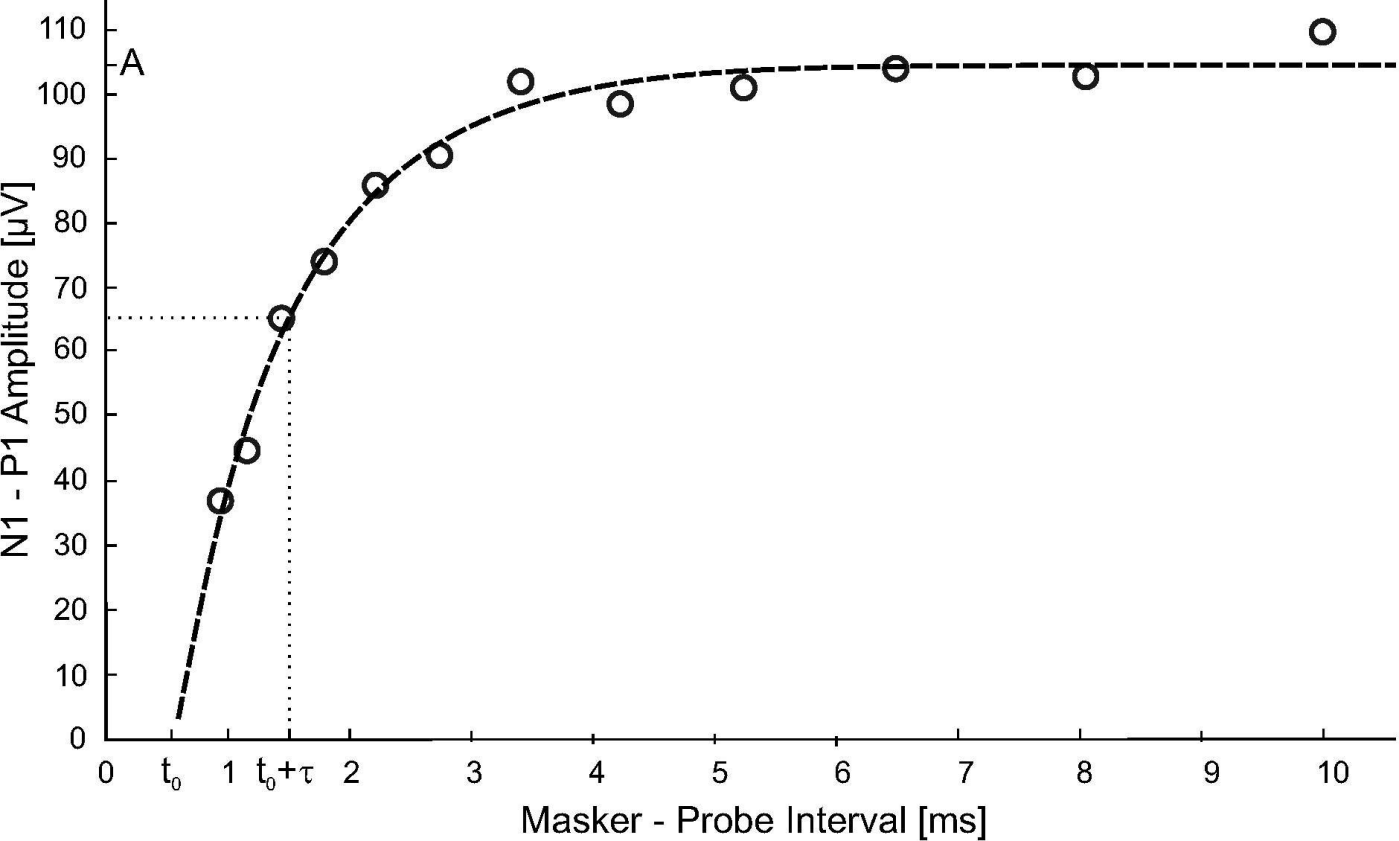
A representative ECAP measurement of subject 2.2 on electrode 5. The fitted exponential function (dashed line) indicates an absolute recovery period *t*_*0*_ = 0.52 ± 0.07 ms, the time constant τ = 1.0 ± 0.1 ms indicates the time needed to reach 63.2% of the maximal amplitude A = 104 ± 2 μV in this measurement.

The measurement of the electrically evoked compound action potential (ECAP) recovery functions was performed using Custom Sound EP software (Cochlear Ltd., Sydney, Australia) with a method described by Miller et al. [26] in subjects taking part in the second experiment. Apical, medial and basal electrodes (5, 12 and 20) were measured. The masker probe interval (MPI) was varied between 300 μs and 10 ms in these measurements. An exponential function shown in Eq (2) was fitted by the Custom Sound EP software to get the saturation level *A*, absolute refractory period *t*_*0*_ and a measure of the relative refractory period *τ* (Fig 4).

$$ECAP(MPI)=A(1-exp\left(\left(t_{0}-MPI\right)/\tau \right).\left[\mu \text{V}\right]$$ (3)

Spearman correlation was calculated between the rank of refractory period measures (*t*_*0*_, τ) and the masking half-life constant *T*_*½*_ which achieved best performance for each individual subject.

### Ethics statement

All procedures were approved by the ethics committee of Hannover Medical School and the study protocol conformed to the declaration of Helsinki. Participants gave written informed consent before data collection.

## Results

**Fig 5:**
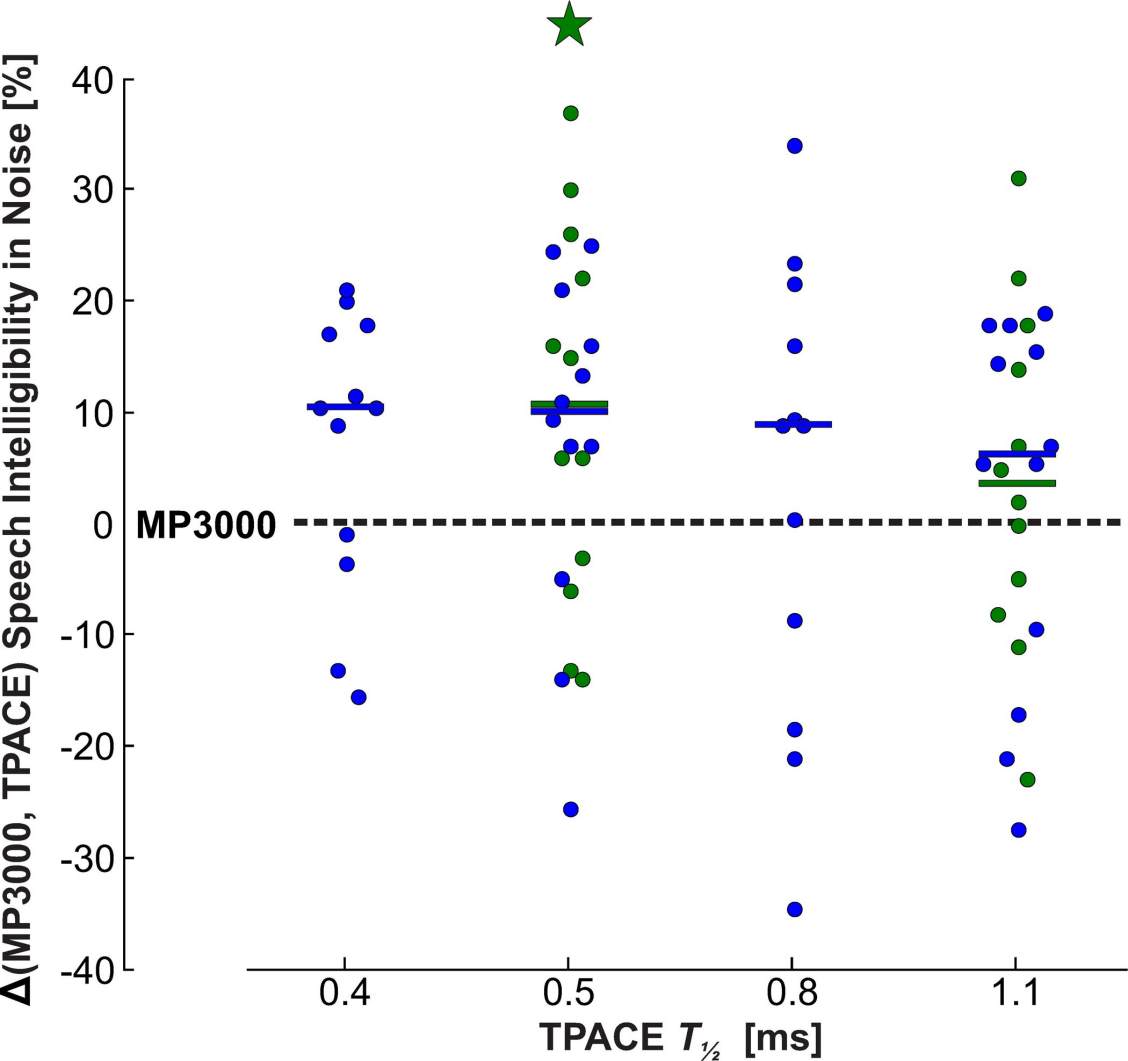
Speech intelligibility performance with different temporal masking half-lifes s using the HSM sentence test in noise. The absolute performance difference between MP3000 and different TPACE conditions is presented for the first (green) and second (blue) study. Each dot shows the averaged result of each subject in the respective condition, the group median is presented as a bar in the respective group color. Speech intelligibility in noise with TPACE *T*_*½*_ = 0.5 ms was significantly better than MP3000 in the first study (p < 0.05; Quade test with three groups). The statistical analysis of the second study using Quade test with five groups did not show a significant difference between conditions.

**Fig 6:**
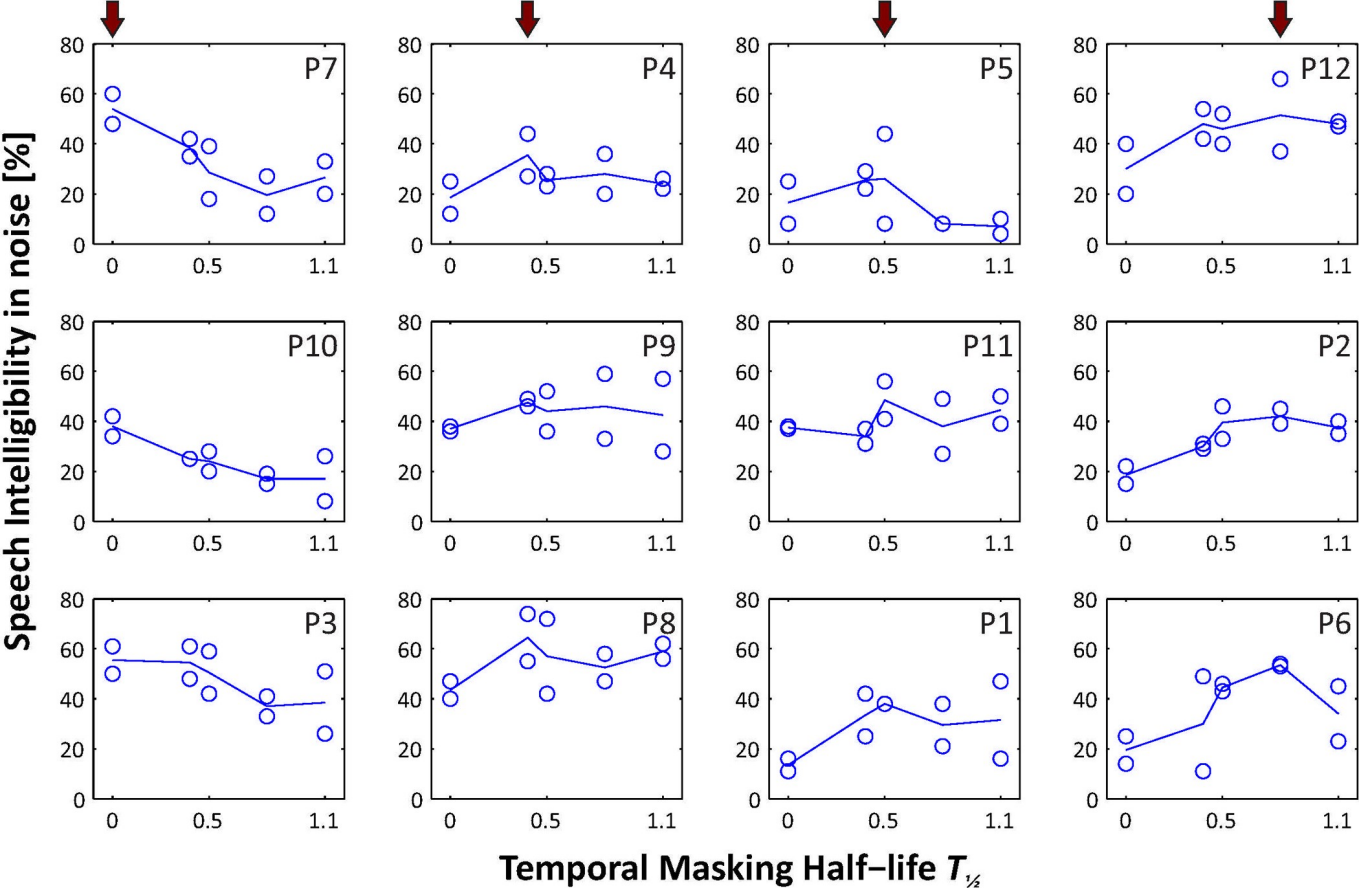
Individual speech intelligibility results of the second study. The blue line presents the average result from two HSM sentence lists (circles) for each temporal masking half-life (0.4, 0.5, 0.8 and 1.1 ms). The SNR for these measurements depends on the performance of the individual patient and ranges from 15 to 5 dB SNR as stated in Table 1. The subjects are ordered in columns according to the temporal masking half-life with the best speech intelligibility (indicated by a red arrow): patients with best results without temporal masking in the first column and patients showing best speech intelligibility with temporal masking constant *T*_*½*_ = 0.8 ms are in the fourth row. No patients showed optimal speech intelligibility with the strongest temporal masking constant of 1.1 ms.

Fig 5 presents the speech intelligibility results as difference scores obtained with MP3000 and TPACE averaged for each subject. Overall, median speech intelligibility in noise was better with TPACE using *T*_*½*_ = 0.5 ms than with MP3000 by 11% in the first (Quade Test of three conditions; significant with p = 0.02) and by 10% (Quade Test of five conditions; not significant with p = 0.3) in the second study (Fig 5). Further increase of the temporal masking half-life *T*_*½*_ led to a decrease of speech intelligibility benefit. However, results of individual speech intelligibility presented in Fig 6, indicate an optimal *T*_*½*_ between 0.4 and 0.8 ms or even without any temporal masking.

**Fig 7:**
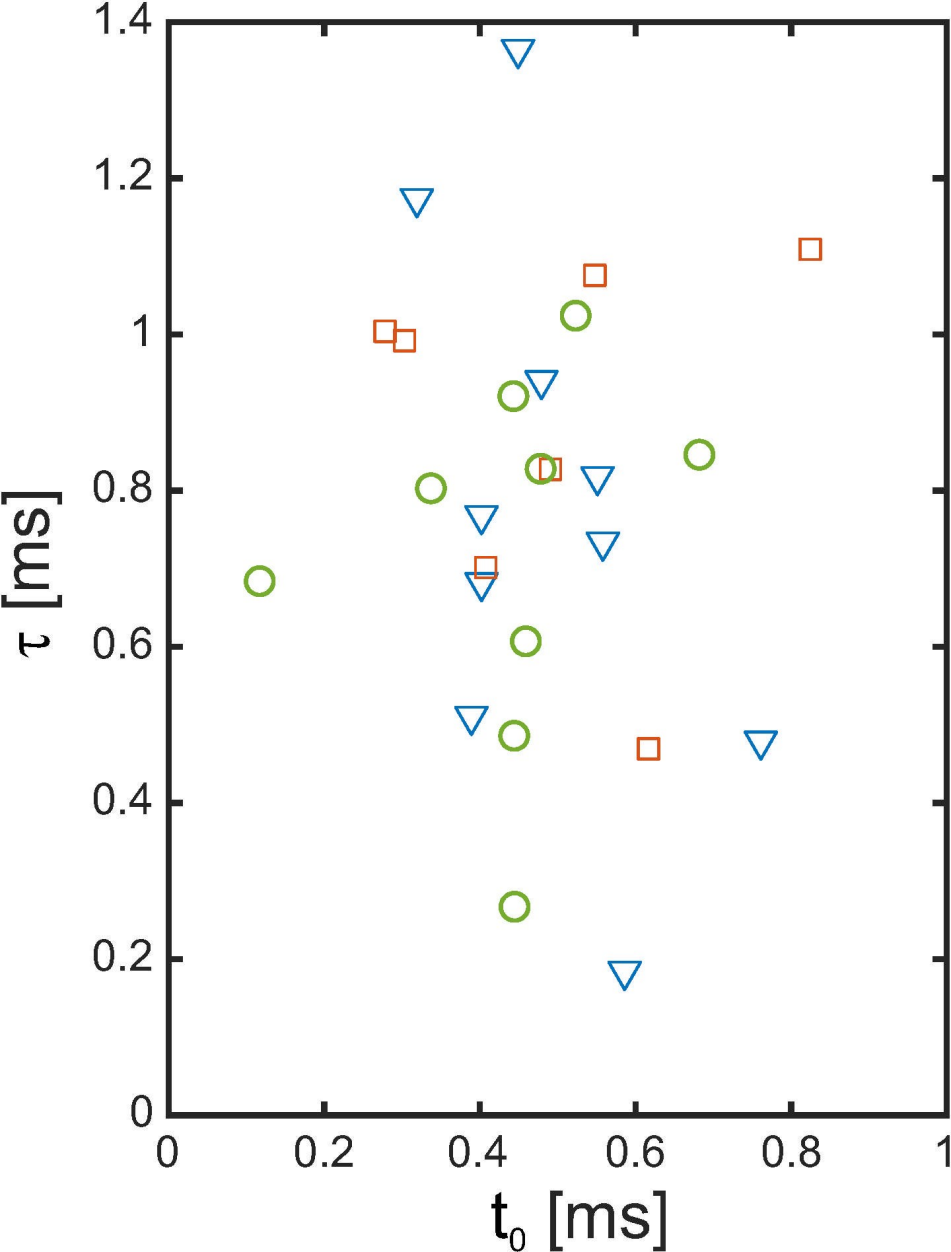
Recovery functions t_0_ and τ of electrode 5 (green circles), electrode 12 (red squares) and electrode 20 (blue triangles).

**Fig 8:**
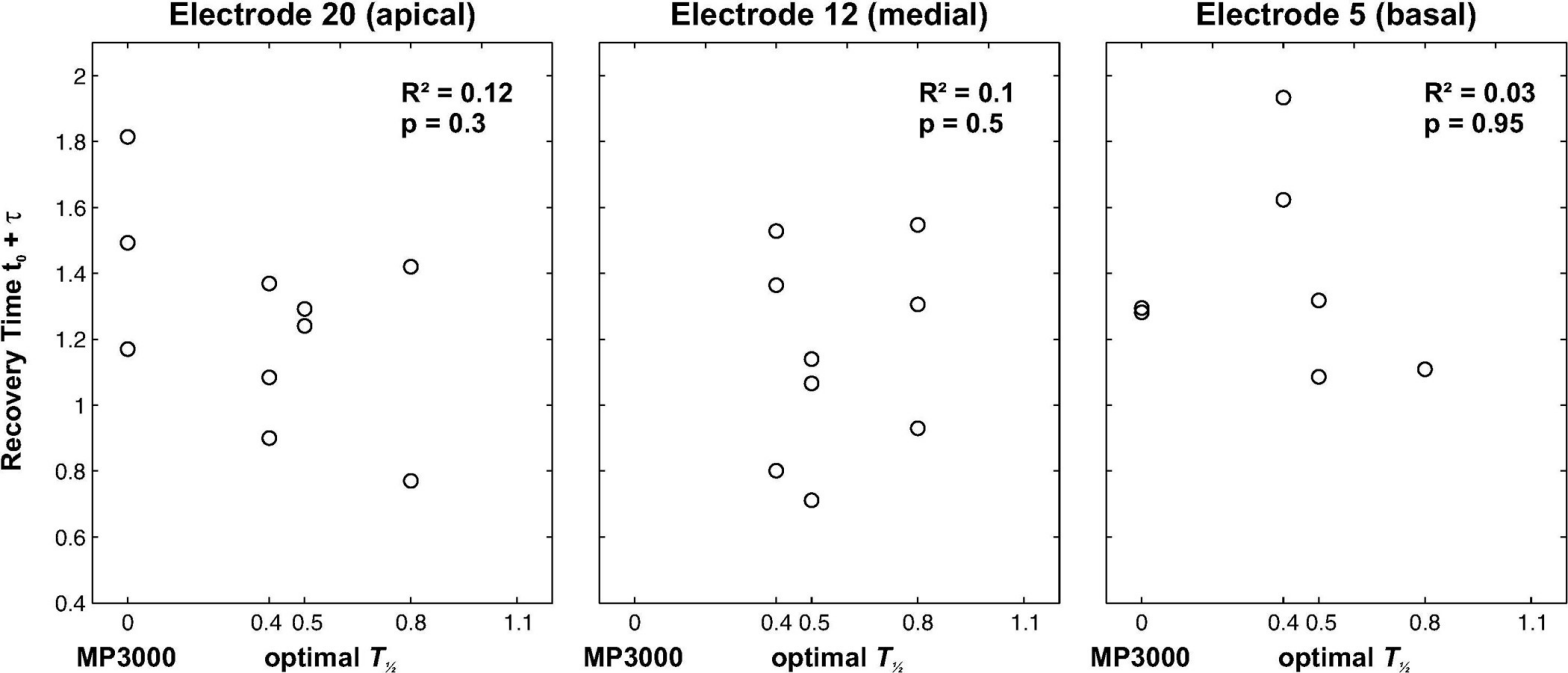
Relation between the ECAP recovery time t_0_ + τ on individual electrodes to the *T*_*½*_ time with the best speech intelligibility performance. Spearman rank coefficient test results (R², p) are reported for each respective plot.

The recovery time constants that were derived from the measurement of the recovery functions and averaged over all three electrodes were *t*_*0*_ = 0.48 ± 0.13 ms and τ = 0.81 ± 0.25 ms. The distribution of the absolute and relative recovery times for individual subjects and electrodes is shown in Fig 7. No significant Spearman’s rank correlation between the individual recovery time constants and the optimal masking half-life constant of the subject was found (Fig 8).

**Fig 9:**
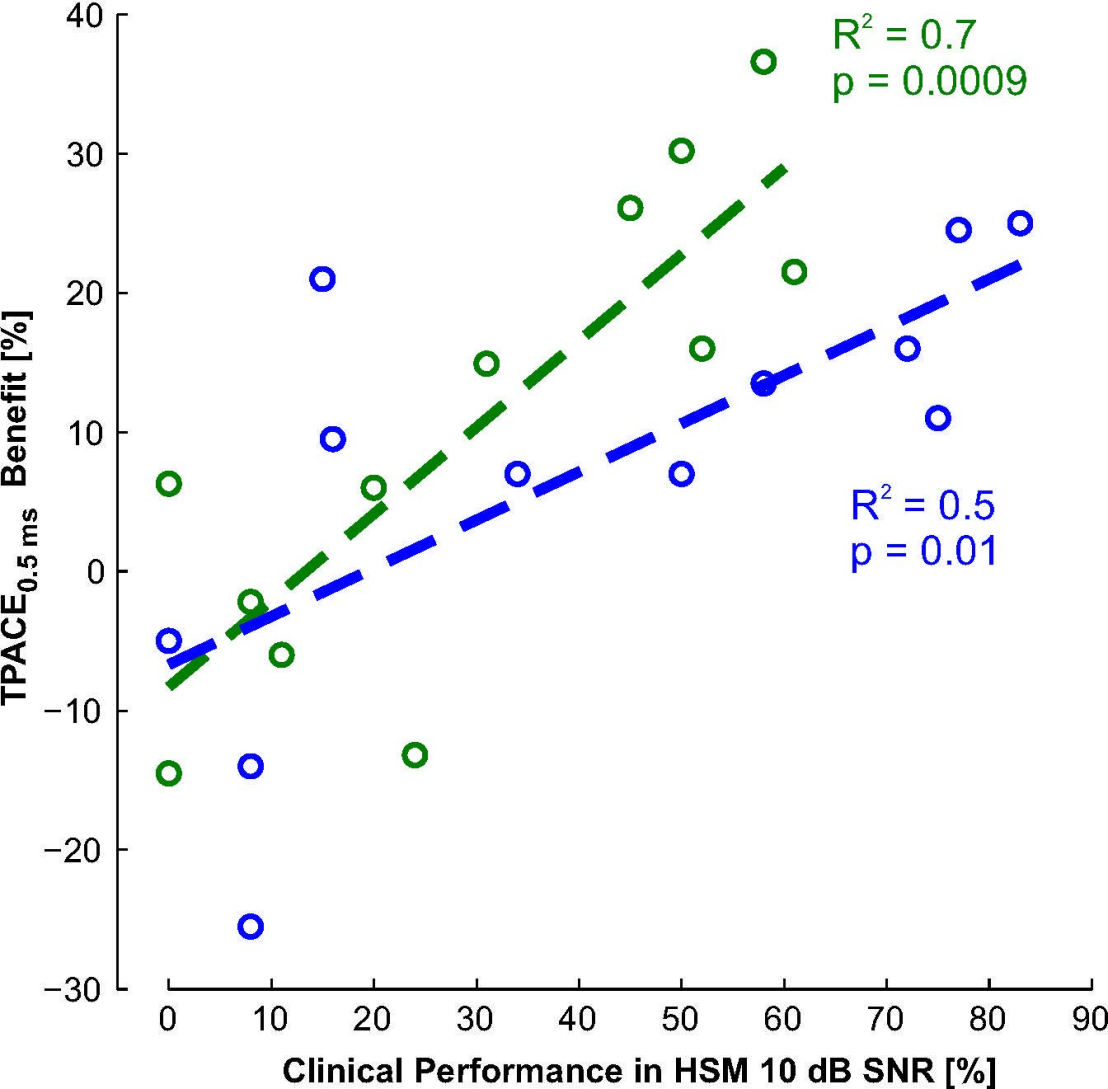
Comparison between the patient performance in the clinical setting (measured with the HSM sentence test at 10 dB SNR during the yearly control visit, Table 1) and the benefit from using TPACE with a *T*_*½*_ of 0.5 ms. The results of the first and second study are presented in green and blue, respectively. Regression between the clinical performance of the subject and the benefit from temporal masking was statistically significant for both studies (R² and p value are shown with the respective color in the plot).

Regression of the speech performance benefit at *T*_*½*_ = 0.5 ms with demographic parameters did not show a significant correlation to the age of the patient, duration of deafness or duration of CI usage. However, regression between the clinical performance of the subjects and their benefit using TPACE with *T*_*½*_ = 0.5 ms (least squares fit; study 1: R² = 0.7 p = 0.0009; study 2: R³ = 0.5, p = 0.01) indicates that subjects with above-average speech intelligibility in their clinical records benefit more from the temporal masking than subjects with worse results during their clinical routine (Fig 9).

## Discussion

This work presented a novel CI sound coding strategy that adds a model of temporal masking to the MP3000 speech coding strategy. The temporal masking model is parameterized through a half-live that mimics the refractory time of the auditory nerve. The results from this study indicate that the incorporation of temporal masking with a half-live *T*_*½*_ of 0.5 ms into the MP3000 sound coding strategy can improve speech intelligibility in noise by 10% (in the second study) to 11% (in the first study) in CI users. This performance improvement can be attributed to the reduction of refractory effects at the level of the auditory nerve achieved by the new sound coding strategy. The TPACE sound coding strategy adds very little complexity to the already low computationally expensive PACE/MP3000.

Previous studies investigated temporal response properties of the human auditory nerve through ECAP responses elicited by each single pulse of a pulse train [27–29]. ECAP responses to each pulse of a pulse train with stimulation rates up to 200 pps presented with similar amplitude, however pulse trains with stimulation rates ranging between 400 to 1000 pps resulted in a maximal response for the first pulse followed by an alternating pattern of weak and strong responses. Further increase of stimulation rates above 2000 pps led to a reduction of the modulation depth in the ECAP amplitude responses. This alternating pattern can be explained by variance in absolute and relative refractory recovery times in ANFs, leading to a non-excitable subpopulation of fibers at the time of the second pulse causing a reduced response to this pulse. Subsequently, the fibers that were not able to respond to the second pulse recover from refractory phase and contribute to a stronger ECAP amplitude response to the following stimulation pulse [29].

The present study used the clinical default stimulation rate of 900 pulses per second for each channel, a stimulation rate reported to produce refractory effects, as explained above. Moreover, it has been shown [13, 30] that the ACE sound coding strategy tends to stimulate with clusters of channels in spectral regions with maximum acoustic energy (e.g. electrodes 20 to 16, 200 to 400 ms, Fig 3A). The simultaneous masking algorithm avoids clustering of stimulated electrodes by masking the neighboring channels around the channel containing the maximum energy (e.g. electrodes 19 and 17, 200 to 400 ms, Fig 3B). Simultaneous masking also leads to stimulation of electrodes outside of the main cluster (e.g. electrode 9, 200 to 400 ms, Fig 3B) leading to more sparse stimulation patterns [13, 30]. Most patients reported a brighter sound when listening to these more sparse excitation patterns created by the PACE sound coding strategy [8].

The addition of a temporal masking model to the simultaneous masking leads to dispersion of stimulation pulses from the channel with the maximum energy to adjacent channels. This effect becomes stronger when increasing the value of the masking half-live (Fig 3C–3H) leading to a shift of stimuli away from the high energy channels towards the adjacent channels which would be completely masked in case the simultaneous masking model (PACE) had been applied alone (Fig 3B). This effect reduces the spatial acuity of the stimulation pattern and leads to an overall reduction of the stimulation rate on the individual channels. This is a possible explanation for the better speech perception obtained with the TPACE speech coding strategy: longer duration between individual pulses gives the neural population more time to recover from their refractory phase and might even avoid possible prolongation of the refractory phase caused by premature stimulation. Since best speech intelligibility performance was achieved with a half-life *T*_*½*_ of 0.5 ms, we can conclude that this amount of temporal masking provides best balance between lower stimulation rate and spatial acuity.

It seems that the consideration of short-acting temporal masking can improve speech intelligibility in CI subjects. The median benefit of TPACE *T*_*½*_ = 0.5 ms was reliably reproduced, even though the second study had more conditions, which–likely due to the multiple comparison correction of five conditions versus only three conditions in the first study–led to non-significant differences in this second trial. Altogether, our data indicate that individual subjects perform best at different masking half-lives. As we focused on temporal masking with half-lives in the range corresponding with the typical absolute and relative refractory period, we anticipated that there might be a correlation between the individual recovery time of the neurons and the optimal *T*_*½*_ value for the individual subjects.

However, the evaluation of the ECAP amplitude recovery functions did not reveal a correlation between this objective measure and the patient-specific optimal temporal masking. It is possible that the recovery functions as described by simple stimuli (a probe and a masker pulse) may not be sufficient to characterize the temporal processing of the auditory nerve caused by complex stimuli such as speech. In turn this may explain a lack of correlation between the measured time constant and the optimal *T*_*½*_ in the TPACE. The use of more sophisticated models of the auditory periphery that additionally incorporate facilitation, spread of excitation and adaptation, as used in the BIC sound coding strategy [21], may be a right step towards obtaining correlations between objective measures and patient-specific fitting parameters.

The evaluation of BIC in eleven CI subjects indicated a significant improvement in the melodic contour identification test, compared to ACE as baseline control. The study failed to show a significant speech intelligibility improvement for the speech in noise test. This result could be explained by a bias of the CI patients to their clinical ACE setting in the acute streaming experiment [21]. This bias was reduced in the current TPACE study by using MP3000 as a control condition. Fourteen out of 24 subjects in the current TPACE study used ACE as clinical strategy, reducing the bias towards their familiar clinical strategy. Still, those patients who had MP3000 as clinical setting were biased towards the MP3000 control condition and not towards TPACE. The study presenting the SAM coding strategy gave only a short anecdotic report on a pilot study with five CI users, claiming considerable benefit especially in pitch identification tasks [20]. The TIPS coding strategy was tested in an acute streaming experiment with eight CI subjects in an acute streaming experiment. The control condition was a custom designed CIS strategy in which all but 8 electrodes were switched off. Even through TIPS performed significantly better than the custom 8-of-8 CIS coding strategy, it is not clear how TIPS performance relates to clinically used strategies like ACE and MP3000.

Overall, the TPACE study is the first CI coding strategy that introduces a temporal masking model and improves speech in noise intelligibility over a clinical coding strategy in two consecutive studies.

Looking at the clinical performance of the tested subjects, a strong correlation between the benefit of TPACE and the performance with the clinically administered speech test material was found. Subjects with good test scores in the clinical aftercare obtained a larger improvement with TPACE over the ACE strategy, than patients with lower scores in the clinical routine with some of them even showing detrimental effects with TPACE. One possible mechanism that could explain this observation might be that poorer performing subjects with an impaired or degraded auditory system are relying on some of the redundant information being discarded by the TPACE strategy. Good performers on the other hand, do benefit from the sparse stimulation of TPACE as their auditory system is still trained on normal perception from acoustic hearing with its according masking phenomena being partially mimicked in TPACE. On the other hand, this observation may also be explained by the fact that the experiment was conducted giving very little time to the CI users to adapt to the new strategy. The generally good performing subjects may be able to adapt faster to the new strategy and gain a benefit from the temporal masking while bad performing subjects are put at a disadvantage by the acute testing scenario.

In the presented study, the number of stimulated channels remained constant over all conditions. Therefore, no significant change in power consumption between TPACE and the MP3000 baseline was expected. However, it has been shown that MP3000/PACE achieves the same speech understanding performance than ACE while reducing the number of selected electrodes for stimulation from 8 to 4. A similar effect would be expected for TPACE. Finally, it is important to mention that reducing the number of selected electrodes in each frame can be used to increase the pulse duration and therefore reduce the current amplitude. Lower current amplitudes allow for a lower supply voltage of the CI potentially yielding further power savings [31].

A real time implementation of the strategy on a clinical behind-the-ear speech processor can reveal if TPACE is able to improve the speech intelligibility in a more realistic setting, especially in subjects with below-average performance in a take-home evaluation with a longer adaptation period.
